# Neuroprotective Effect of AM404 Against NMDA-Induced Hippocampal Excitotoxicity

**DOI:** 10.3389/fncel.2019.00566

**Published:** 2019-12-20

**Authors:** Soraya Wilke Saliba, Tiziana Bonifacino, Tsvetan Serchov, Giambattista Bonanno, Antônio Carlos Pinheiro de Oliveira, Bernd L. Fiebich

**Affiliations:** ^1^Neuroimmunology and Neurochemistry Research Group, Department of Psychiatry and Psychotherapy, Medical Center—University of Freiburg, Faculty of Medicine, University of Freiburg, Freiburg, Germany; ^2^Unit of Pharmacology and Toxicology and Center of Excellence for Biomedical Research, Department of Pharmacy, School of Medical and Pharmaceutical Sciences, University of Genoa, Genoa, Italy; ^3^Laboratory of Stereotaxy and Interventional Neuroscience, Department of Stereotactic and Functional Neurosurgery, Medical Center—University of Freiburg, Faculty of Medicine, University of Freiburg, Freiburg, Germany; ^4^Istituto di Ricovero e Cura a Carattere Scientifico (IRCCS), Ospedale Policlinico San Martino, Genoa, Italy; ^5^Department of Pharmacology, Universidade Federal de Minas Gerais, Belo Horizonte, Brazil

**Keywords:** AM404, hippocampus, neuroinflammation, excitotoxicity, LPS, NMDA, cannabinoid receptor, vanilloid receptor

## Abstract

Different studies have demonstrated that inflammation and alterations in glutamate neurotransmission are two events contributing to the pathophysiology of neurodegenerative or neurological disorders. There are evidences that N-arachidonoylphenolamine (AM404), a cannabinoid system modulator and paracetamol metabolite, modulates inflammation and exerts neuroprotective effects on Huntington’s (HD) and Parkinson’s diseases (PD), and ischemia. However, the effects of AM404 on the production of inflammatory mediators and excitotoxicity in brain tissue stimulated with N-methyl-D-aspartic acid (NMDA) are not elucidated. In this present study, we investigated the effects of AM404 on the production of inflammatory mediators and neuronal cell death induced by NMDA in organotypic hippocampal slices cultures (OHSC) using qPCR, western blot (WB), and immunohistochemistry. Moreover, to comprehend the mechanism of excitotoxicity, we evaluated the effects of AM404 on glutamate release in hippocampal synaptosomes and the NMDA-induced calcium responses in acute hippocampal slices. Our results showed that AM404 led to a significant decrease in cell death induced by NMDA, through a mechanism possibly involving the reduction of glutamate release and the calcium ions responses. Furthermore, it decreased the expression of the interleukin (IL)-1β. This study provides new significant insights about the anti-inflammatory and neuroprotection effects of AM404 on NMDA-induced excitotoxicity. To understand the effects of AM404 in these processes might contribute to the therapeutic potential of AM404 in diseases with involvement of neuroinflammation and neurodegeneration and might lead to a possible future treatment of neurodegenerative diseases.

## Introduction

The role of excitotoxicity in the etiology or progression of several human neurodegenerative disorders such as Alzheimer’s (AD), Parkinson’s (PD) and Huntington’s (HD) diseases, epilepsy or amyotrophic lateral sclerosis (ALS) has been proposed (Palop et al., 2006; Dong et al., 2009). Excitotoxicity is a pathologic process characterized by the increase of calcium ions influx through mainly N-methyl-d-aspartic acid (NMDA) receptors that result in both, an increase of glutamate release and activation of many enzymes culminating in neuronal cell death (Choi, 1988; Waxman and Lynch, 2005). A promising target for the therapeutic intervention of several progressive neurodegenerative diseases is the modulation of the endocannabinoid system (Scotter et al., 2010). Some studies have suggested that the endocannabinoid system plays a protective role against excitotoxic damage (Marsicano et al., 2003; Mechoulam, 2003; Veldhuis et al., 2003), mostly *via* cannabinoid CB1 receptor inhibiting N-type Ca^2+^ channels activity and consequently reduces glutamatergic transmission (Shen et al., 1996; Lévénès et al., 1998; van der Stelt et al., 2002). The CB1 receptor is the most abundant G protein-coupled receptor in the brain (Howlett et al., 1990, 2010) and it is expressed on glutamatergic and GABAergic neurons in brain regions such as the hippocampus, cortex, and basal ganglia (Tsou et al., 1998; Mackie, 2005). Besides CB1 and CB2 receptors, cannabinoid agonists also activate transient receptor potential vanilloid type 1 (TRPV1; Smart et al., 2000; Ross, 2003). TRPV1 is a non-selective cation channel and similarly expressed in numerous regions in the brain, including cortex, hippocampus, and corpus callosum (Tóth et al., 2005).

N-arachidonoylphenolamine (AM404), a paracetamol metabolite, blocks the anandamide membrane transporter (AMT; Beltramo et al., 1997; Giuffrida et al., 2000) and is an agonist of TRPV1 (De Petrocellis et al., 2000; Zygmunt et al., 2000) and CB1 receptors (Khanolkar et al., 1996; Beltramo et al., 2000; Mitchell et al., 2007). The neuroprotective effects of AM404 on some neurodegenerative models through the activation of the CB1 or/and TRPV1 receptors has been demonstrated. In a rat model of HD induced by the injection of 3-nitropropionic acid, AM404 was able to attenuate the hyperkinetic signs and recover neurochemical (GABA and dopamine) deficits (Lastres-Becker et al., 2002) *via* TRPV1 receptor (Lastres-Becker et al., 2003). However, on an ischemia-induced neuronal injury, AM404 protected CA1 layer neurons of the hippocampus through CB1 and opioid receptors but not involving TRPV1, and prevented ischemia-induced memory impairment (Zani et al., 2007).

AM404 ameliorates parkinsonian effects induced by 6-hydroxydopamine in rats (Fernandez-Espejo et al., 2004) and recovered the dopamine depletion and tyrosine hydroxylase deficit, probably by an antioxidant effect (García-Arencibia et al., 2007). In this model of 6-OHDA, enhanced glutamatergic transmission after DA depletion has been shown and AM404 was able to reduce the frequency of glutamatergic spontaneous activity and SR141716 (CB1 antagonist) but not capsazepine (TRPV1 antagonist) blocked this effect (Gubellini et al., 2002). Moreover, AM404 has been described to attenuate seizures from epilepsy models using pentylenetetrazole (PTZ; Manna and Umathe, 2012) or kainic acid (Shubina et al., 2015, 2017). Manna and Umathe (2012) further demonstrated that in an epilepsy model using PTZ, the protective effects of AM404 involved CB1 but not TRPV1 receptors.

The effects of AM404 on the excitotoxicity and production of inflammatory mediators in brain tissue stimulated with NMDA are not elucidated. Thus, in this current study, we evaluated if AM404 is able to prevent NMDA-induced excitotoxicity and inflammation by evaluating cell death and inflammatory parameters in organotypic hippocampal slices cultures (OHSC), glutamate release in synaptosomes, and intracellular calcium responses in acute hippocampal slices stimulated with NMDA.

## Materials and Methods

### Ethics Statement

The experiments were performed using neonatal female and male C57BL/6 wild-type (WT). Neonatal mice pups were obtained from Center for experimental models and transgenic services (CEMT, Freiburg) and used in accordance with the German animal welfare law for the use of experimental animals (approved protocol No. X-13/06A by the Regierungspräsidium Freiburg).

### Drugs

AM404 (Alomone Labs) was dissolved in the physiological medium for the synaptosome experiment and in DMSO for the other experiments (Merck KGaA, Darmstadt, Germany). NMDA was resuspended in Dulbecco’s phosphate-buffered saline (DPBS; Gibco^®^ by Life Technologies, Germany) as 100 mM stock, and was used at a final concentration of 10–50 μM in the OHSC. Lipopolysaccharide (LPS) from *Salmonella typhimurium* was resuspended in DPBS as 5 mg/ml stock and was used at a final concentration of 10 ng/ml or 100 ng/ml. Solvent concentrations in the culture media were maintained at 0.1%.

### Preparation of Organotypic Hippocampal Slice Cultures (OHSC)

As previously described (Saliba et al., 2017a), OHSCs were prepared from 2 to 3 days old C57BL/6 WT mice. Animals were decapitated, the hippocampi were dissected and placed in a chopper for the preparation of the slices with a thickness of 350 μM. The integral slices were selected and transferred to a 0.4 μM culture plate inserts (Millipore). The inserts were placed in a 6-well culture plate containing 1 ml of OHSC medium [0.5× minimum essential medium (MEM) containing Earl’s salts, 25% horse serum, 25% basal medium (BME) without glutamate and containing Earl’s salts, 2 mM glutamax, and 0.35% glucose]. Then, the plate was incubated at 35°C in a humidified atmosphere with 5% CO_2_ and the culture medium was changed after the first day of preparation following every 2 days.

### Quantification of Neuronal Cell Death in OHSC

After 7 days in culture, OHSCs were pre-treated with AM404 (10, 25, or 50 μM) for 30 min and NMDA (25 μM) was added for additional 4 h. The slices were then washed with 37°C DPBS and the media replaced with NMDA-free medium containing 5 μg/ml propidium iodide (PI, Sigma), and incubated for 24 h. Thereafter, OHSCs were washed with cold DPBS followed by 4% PFA incubation for 1 h. After fixation, the slices were washed with DPBS and incubated with 5% normal goat serum (NGS) in PBS containing 0.3% Triton X-100 (PBS^+^) for at least 2 h. Then, slices were incubated overnight with an anti-mouse-NeuN-488 (1:1,000) in 1% NGS/PBS^+^ at 4°C. Analyses of the slices were done by confocal imaging using a Zeiss microscope (Zeiss, Oberkochen, Germany) from Life Imaging Center (LIC, Center for Biological Systems Analysis, Freiburg, Germany). Quantification of fluorescence intensity was done using ImageJ software.

### Synaptosome Purification

Synaptosomes were prepared from C57BL/6 WT mouse hippocampus as described (Raiteri et al., 2008). The tissue was homogenized in 0.32 M sucrose, buffered at pH 7.4. The homogenate was centrifuged (5 min, 1,000× *g* at 4°C) and the supernatant again centrifuged at 12,000× *g* for 10 min. The pellet was resuspended in Tris-buffered 0.32 M sucrose, layered on a discontinuous Percoll^®^ gradient (2, 6, 10, and 20% v/v in Tris-buffered 0.32 M sucrose), and centrifuged at 33,500× *g* for 5 min. The layer between 10 and 20% Percoll^®^ was collected, washed and centrifuged at 20,000× *g* for 15 min. The pellet was resuspended in physiological medium having the following composition (mM): NaCl, 140; KCl, 3; MgSO_4_, 1.2; NaH_2_PO_4_, 1.2; NaHCO_3_, 5; CaCl_2_, HEPES, 10; glucose, 10; pH 7.4.

### Release Experiments

Synaptosomes were incubated (15 min, 37°C) with 0.05 μM [^3^H]D-Aspartate ([^3^H]D-Asp), a non-metabolizable analog of Glu which labels the intra-terminal releasable pools of the excitatory amino acid (Fleck et al., 2001; Raiteri et al., 2007). Aliquots were distributed on microporous filters placed at the bottom of a set of 24 parallel superfusion chambers maintained at 37°C (Superfusion System, Ugo Basile, Comerio, Varese, Italy) and superfused as described (Milanese et al., 2010). Superfusion was started with a physiological medium at a rate of 0.5 ml/min and continued for 51 min. After 36 min of superfusion, to equilibrate the system, six samples (*t* = 33–36; *t* = 36–39; *t* = 39–42; *t* = 42–45; *t* = 45–48; *t* = 48–51) were collected. NMDA (30 μM) plus glycine (1 μM) was added at *t* = 39 min; and AM404 (0.1, 1 or 50 μM) at *t* = 30 and maintained until the end of the experiment. Then, samples were collected and superfused synaptosomes were counted for radioactivity. Tritium released in each sample was calculated as fractional rate × 100 (percentage of the total synaptosomal neurotransmitter content at the beginning of the respective collection period). Drug effects were evaluated by calculating the ratio between the efflux in the fourth sample collected (in which the maximum effect of NMDA was generally reached) and the efflux of the second sample collected (basal efflux). This ratio was compared to the corresponding ratio obtained under control conditions. Appropriate ratios in each experiment were compared to evaluate the AM404 effect.

### Calcium Imaging

Acute brain slices of C57BL/6 WT mice were prepared as described (Dawitz et al., 2011; Holz et al., 2019). In brief, 6 days old animals were decapitated and the forebrain was removed and replaced in carbogenated (5% CO_2_ and 95% O_2_) ice-cold artificial cerebrospinal fluid (ACSF; 125 mM NaCl, 25 mM NaHCO_3_, 27 mM Glucose, 2.5 mM KCl, 1.25 mM NaH_2_PO_4_, 1 mM MgCl_2_ and 2 mM CaCl_2_). Four to six coronal slices (300 μm thickness) containing the hippocampus area were cut by using a vibratome (DTK-1000, Dosaka, Japan). The slices were collected and recovered at room temperature (RT) for 30 min in carbogenated ACSF. In a chamber containing 1 ml of carbogenated ACSF, the hemispheres were separated and incubated with 50 mM Fura-2 acetoxymethyl ester (Invitrogen) in the presence of 0.1% pluronic acid (Invitrogen), for 20–30 min at 35°C protected from the light. After incubation, the slices were rinsed in carbogenated ACSF and incubated with 50 μM AM404 for 30 min. One slice was placed in the imaging chamber and fixed with a metal harp superfused with carbogenated ACSF containing 1.5 mM MgCl_2_ and 1.6 mM CaCl_2_. The fluorescence imaging was taken at the light microscope (Axioskop FS2, Zeiss; 40× water immersion objective, Olympus Optical, Tokyo, Japan) using the TILLvisION program. Single Fura-2-fluorescent neurons were selected as regions of interest and the baseline fluorescence signal (F_0_) was recorded for at least 30 s before NMDA. The changes on the fluorescence signals (ΔF) were calculated with the formula (F-F_0_/F_0_) where F is fluorescence intensity and F_0_ is baseline fluorescence intensity. The area under the curve was determined for 90 s after NMDA.

### qPCR Analysis

After 7 days in culture, OHSCs were pre-treated with AM404 (10, 25 or 50 μM) for 30 min and NMDA (25 μM) was added for an additional 4 h. Thereafter, OHSCs were washed with cold DPBS followed the mRNA isolation was performed using the GeneMATRIX Universal RNA Purification Kit from Roboklon, according to the manufacturer’s protocol. After the isolation, 500 ng RNA were mixed with 2 μg of random hexamer oligonucleotides in a 30 μl total reaction volume and denatured at 70°C (10 min). The synthesized cDNA was used for the real-time PCR amplification that was carried out using the CFX96 real-time PCR detection system (Bio-Rad Laboratories GmbH, Munich, Germany). The primers (table below) were designed by using Universal Probe Library Assay Design Center.

**Table 1:** 

Gene	Forward primer (5′–3′)	Reverse primer (5′–3′)
CD11b	TACCGTCTACTACCCATCTGGC	TTGGTGAGCGGGTTCTGG
CSF1R	CGAGGGAGACTCCAGCTACA	GACTGGAGAAGCCACTGTCC
GAPDH	TGGGAAGCTGGTCATCAAC	GCATCACCCCATTTGATGTT
GFAP	GGAGGTGGAGAGGGACAAC	GTTTCATCTTGGAGCTTCTGC
Iba-1	CAGGGATTTGCAGGGAGGAAA	AGTTTGGACGGCAGATCCTC
IL-1β	TGTGATGAAAGACGGCACAC	CTTCTTCTTTGGGTATTGTTTGG
IL-6	CCTGGAGTTTGTGAAGAACAACT	GGAAGTTGGGGTAGGAAGGA
iNOS	CTTTGCCACGGACGAGAC	TCATTGTACTCTGAGGGCTGAC
mPGES-1	GCACACTGCTGGTCATCAAG	ACGTTTCAACGCGTCCTC
TNFα	CCCACGTCGTAGCAAACCACCA	CCATTGGCCAGGAGGGCGTTG

### Western Blot (WB)

After 7 days in culture, OHSCs were pre-treated with AM404 (10, 25 or 50 μM) for 30 min and NMDA (25 μM) was added for an additional 4 h. The slices were then washed with 37°C DPBS and the media replaced with NMDA-free medium and incubated for 24 h. Thereafter, OHSCs were washed with cold DPBS and lysed in the lysis buffer (42 mM Tris-HCl, 1.3% sodium dodecyl sulfate, 6.5% glycerin, 100 μM sodium orthovanadate, and 2% phosphatase and protease inhibitors; Saliba et al., 2017a). The protein concentration of the samples was measured using the bicinchoninic acid (BCA) protein assay kit (Thermo Fisher Scientific, Bonn, Germany) according to the manufacturer’s instructions. For western blot (WB), 10–20 μg of total protein from each sample were subjected to sodium dodecyl sulfate-polyacrylamide gel electrophoresis (SDS-PAGE) under reducing conditions. Afterward, proteins were transferred onto polyvinylidene fluoride (PVDF) membranes (Merck Millipore, Darmstadt, Germany) by semi-dry blotting. After blocking with Roti-Block (Roth, Karlsruhe, Germany), membranes were incubated overnight with primary antibodies: goat anti-COX-2 (1:500; Santa Cruz Biotechnology, Heidelberg, Germany), and rabbit anti-actin (1:5,000; Sigma Aldrich). The proteins were detected with horseradish peroxidase-coupled rabbit anti-goat IgG (Santa Cruz, 1:100,000 dilution) and goat anti-rabbit IgG (GE Healthcare, 1:25,000 dilution) using enhanced chemiluminescence (ECL) reagents (GE Healthcare, Freiburg, Germany). Densitometric analysis was performed using ImageJ software (NIH, Bethesda, MD, USA), and β-actin control was used to confirm equal sample loading and normalization of the data.

### Data Analysis

The results were presented as mean ± SEM. Data were analyzed using one-way analysis of variance (ANOVA) followed by Newman–Keuls post-test. The level of statistical significance was considered as *p < 0.05, **p < 0.01, ***p < 0.001. Graph Pad Prism (Graph Pad Software, San Diego, CA, USA) was used for performing all statistical analysis.

Data for synaptosomes are expressed as mean ± SEM and *p*-value < 0.05 was considered significant. Multiple comparisons were performed using the ANOVA followed by Bonferroni *post hoc* test. Analyses were performed by SigmaStat (Systat Software, Inc., San Jose, CA, USA) software.

Data for calcium imaging were expressed as fluorescence intensity (ΔF/F baseline) vs. time and calculated the area under the curve (AUC). To compare the groups, unpaired *t*-test was evaluated using Graph Pad Prism (Graph Pad Software, San Diego, CA, USA).

## Results

### AM404 Prevented the NMDA-Induced Neuronal Toxicity in OHSC

**Figure 1:**
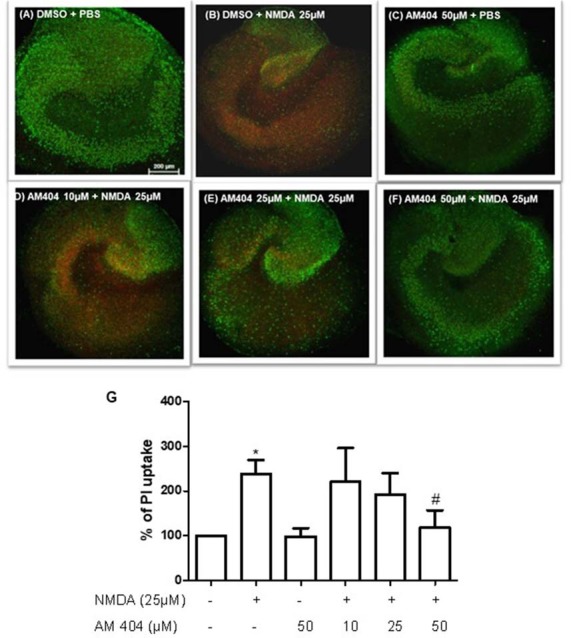
Effects of N-arachidonoylphenolamine (AM404) on excitotoxicity in mouse hippocampal slices cultures [*n* = 3 organotypic hippocampal slices cultures (OHSCs)/group]. **(A–F)** Representative confocal images of the immunostaining with propidium iodide (PI; red) and with the neuronal nuclear marker NeuN (green). **(G)** Quantitative analysis of the PI fluorescence. The results are expressed as mean ± SEM. *p < 0.05 with respect to negative control and ^#^*p* < 0.05 compared with N-methyl-D-aspartic acid [NMDA; 25 μM; one-way analysis of variance (ANOVA), Newman–Keuls test].

We studied whether AM404 has a neuroprotective effect on excitotoxicity induced by NMDA in OHSC. As shown in Figure 1B, the intensity of PI uptake was increased in OHSC after NMDA (25 μM) stimulation compared with negative control (Figure 1A). Incubation of OHSC with 10 or 25 μM of AM404 did not alter the intensity of PI uptake (Figures 1D,E,G). However, pre-treatment with 50 μM of AM404 (Figures 1F,G) potently prevented the increase of PI uptake levels and thus excitotoxicity induced by NMDA back to basal levels (Figures 1A,C).

### AM404 Reduced [^3^H]D-Asp Release in Hippocampal Synaptosomes and the NMDA-Induced Calcium Responses in Acute Hippocampal Brain Slices

**Figure 2:**
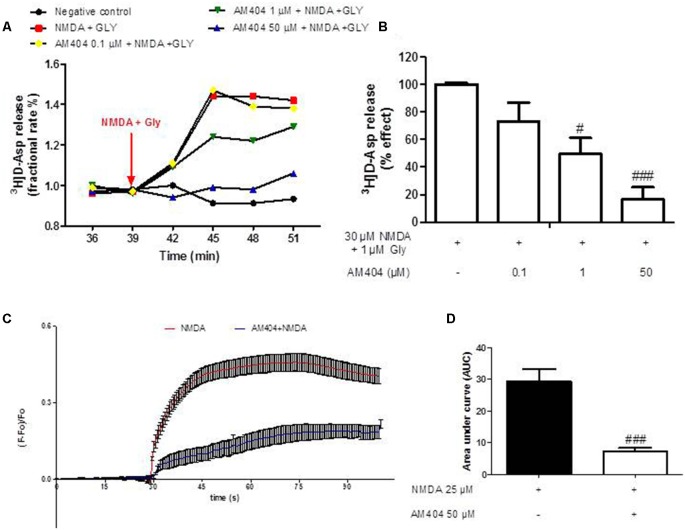
Effects of AM404 on [^3^H]D-Asp release in hippocampal synaptosomes and intracellular Ca^2+^ responses. **(A)** Time course of [^3^H]D-Asp release induced by NMDA plus Gly and effects of AM404 in hippocampal synaptosomes. Results are expressed as fractional rate percent. **(B)** AM404 inhibition of the NMDA plus Glycine-evoked-[^3^H]D-Asp release in hippocampal synaptosomes. **(C)** Data of calcium imaging expressed as fluorescence intensity (ΔF/F baseline) vs. time. **(D)** Data of calcium imaging calculated the area under the curve (AUC). Data are means ± SEM of three independent experiments. ^#^*p* < 0.05 and ^###^*p* < 0.001 compared to NMDA (one-way ANOVA followed by Bonferroni *post hoc* tests for [^3^H]D-Asp release and unpaired *t*-test of three independent experiments for calcium imaging).

In order to investigate whether AM404 might modulate glutamate release and calcium responses contributing to its neuroprotective effect, we evaluated the effect of AM404 on [^3^H]D-Asp release induced by NMDA plus glycine (Gly) in hippocampal synaptosomes and calcium imaging in OHSC. As shown in Figure 2A, NMDA (30 μM) plus Gly (1 μM) increased [^3^H]D-Asp release and this effect reached the maximal value starting from *t* = 45 min of superfusion. AM404 (0.1, 1 and 50 μM) strongly and concentration-dependently reduced NMDA plus Gly-evoked-[^3^H]D-Asp release by about 30%, 51%, and 87%, respectively (Figure 2B). In addition, NMDA increased the calcium ions responses, which was prevented by AM404 (50 μM; Figures 2C,D).

### AM404 Prevented NMDA-Induced Increase of IL-1β Expression but Did Not Affect the NMDA Mediated Expression of IL-6, TNFα, mPGES-1, and iNOS

**Figure 3:**
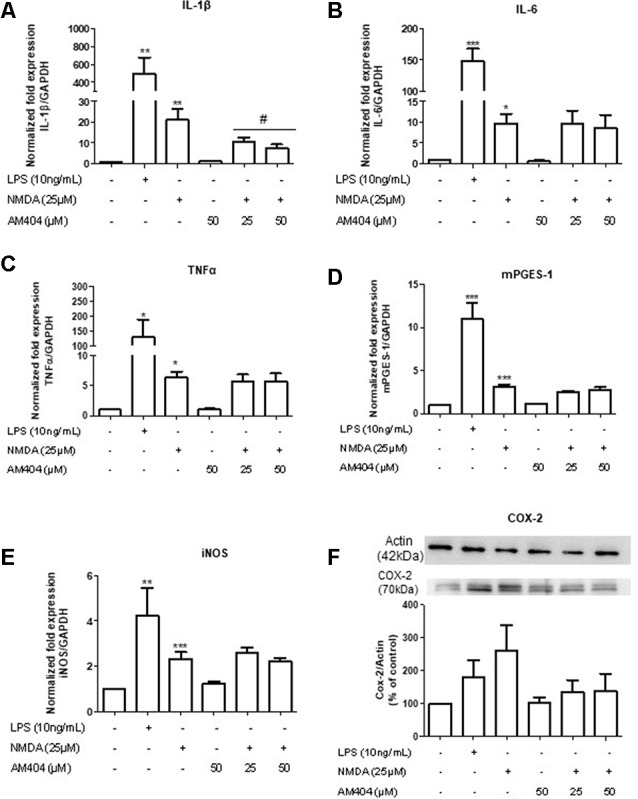
Effects of AM404 on NMDA-induced inflammatory mediators in organotypic hippocampal slices cultures (OHSC). OHSCs were pre-treated with two concentrations of AM404 (25 or 50 μM) for 30 min before stimulating with 10 ng/ml LPS or 25 μM NMDA. After 4 h, interleukin (IL)-1β **(A)**, IL-6 **(B)**, TNFα **(C)**, mPGES-1 **(D)**, and iNOS **(E)** were measured by PCR. After 24 h, COX-2 **(F)** was evaluated by western blot (WB). Data are expressed as mean ± SEM of at least three OHSCs/group. *p < 0.05, **p < 0.01 and ***p < 0.001 with respect to negative control and ^#^*p* < 0.05 in comparison to 25 μM NMDA (one-way ANOVA followed by the Newman–Keuls post-test).

The excitotoxicity process is directly correlated with inflammation. To confirm this, we first evaluated the effects of different concentrations of NMDA on the expression of inflammatory mediators in OHSC. As shown in Supplementary Figure S1, NMDA (25 μM) increased all inflammatory parameters tested. To investigate the effects of AM404 on inflammation induced by excitotoxicity, we stimulated the slices with 25 μM of NMDA for 4 h. LPS (10 ng/ml) was used as a positive control. As expected, LPS and NMDA statistically increased inflammatory mediators (Figures 3A–E). The pre-treatment with AM404 statistically prevented NMDA-induced IL-1β expression (Figure 3A) and tended towards a decrease of COX-2 protein (Figure 3F). AM404 did not affect the levels of the other cytokines, mPGES-1, and iNOS.

### AM404 Did Not Alter the mRNA Expression of Microglia and Astrocytes Markers

**Figure 4:**
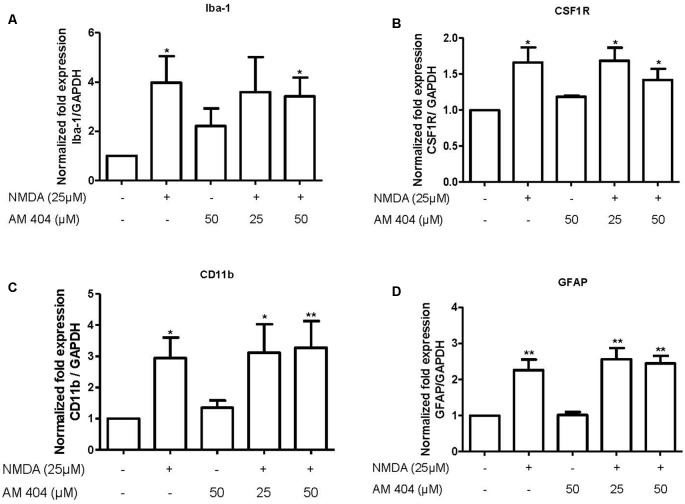
Effect of AM404 on microglia and astrocyte after 25 μM NMDA in OHSC. The OHSCs were pre-treated with two concentrations of AM404 (25 or 50 μM) for 30 min before stimulating with 25 μM NMDA. After 4 h, microglia markers, Iba-1 **(A)**, CSF1R **(B)** and CD11b **(C)**, an astrocyte marker, GFAP **(D)** were measured by qPCR. Data are expressed as mean ± SEM of at least three OHSCs/group. *p < 0.05, **p < 0.01 with respect to negative control (one-way ANOVA followed by the Newman–Keuls post-test).

The main source for neuroinflammatory mediators are immune cells. Thus, we next investigated the effect of AM404 to modulate the activation of microglia and astrocytes. As demonstrated in Figure 4, NMDA statistically increased the expression of microglia (Figures 4A–C) and astrocyte markers (Figure 4D). However, the pre-treatment with AM404 did not alter these effects.

## Discussion

In this study, we have demonstrated that NMDA increased cell death in OHSC and AM404 prevented this effect in a concentration-dependent manner through a mechanism possibly involving the decrease of glutamate release in hippocampal synaptosomes and intracellular calcium responses in hippocampal slices. The cannabinoid modulator/paracetamol metabolite also reduced the production of IL-1β, an important mediator associated with neurodegenerative and neuroinflammatory conditions.

In accordance with our results, some studies have shown neuroprotective effects of AM404 on different models. AM404 has been found to protect against neuronal death in an animal model of ischemia (Zani et al., 2007) and epilepsy (Shubina et al., 2017). Huang et al. (2019) demonstrated the treatment with AM404 significantly induced neuroprotection in hippocampal neuronal culture. Moreover, the combination of AM404 and AM374 (Fatty acid amide hydrolase, FAAH, inhibitor) protected against excitotoxicity induced by AMPA (Karanian et al., 2005).

The effects of AM404 on calcium entry have been demonstrated contradictory, depending on the cell type used. AM404 decreased calcium influx in hippocampal neurons (Hampson et al., 2011; Nazıroğlu et al., 2019). Moreover, AM404 directly inhibited the function of L-type voltage-dependent Ca^2+^ channels in rat myotubes (Alptekin et al., 2010) and weakly inhibited Cav3.2 T-type calcium channel in mouse supraspinal (Kerckhove et al., 2014). In contrast, it has been described that AM404 increased the influx of intracellular calcium in a concentration-dependent manner in human MG63 osteosarcoma cells (Chang et al., 2008).

The activation of NMDA receptors increased cytokines which contribute to neurodegeneration (Kaindl et al., 2008). Thus, we evaluated the effects of NMDA receptor activation on the production of inflammatory mediators in OHSCs. We first verified that mRNA expression of some inflammatory parameters (IL-1β, IL-6, TNFα, mPGES-1, iNOS, and COX-2) is induced by NMDA and we show here that the pre-treatment of AM404 prevented the increase on IL-1β expression and had the tendency to decrease COX-2 protein levels induced by NMDA. So far, we are not able to explain why AM404 is only able to reduce IL-1β levels. Possible mechanism might be, that AM404 targets specific signaling pathways involved in IL-1β expression or affects the stability of IL-1β mRNA but not of the other genes investigated.

The direct correlation between IL-1β and excitotoxicity has been described (Fogal and Hewett, 2008) and its involvement in neurodegeneration has been observed in different neurological disorders such as epilepsy (Vezzani and Baram, 2007) and multiple sclerosis (Rossi et al., 2014). IL-1β enhanced NMDA receptor-mediated intracellular calcium and neuronal death (Viviani et al., 2003) and the increase in IL-1β signaling enhanced glutamate-mediated synaptic excitability and neurotoxicity (Rossi et al., 2012). Moreover, an interaction between IL-1β and the endocannabinoid system has been described, based on the evidences that IL-1β increased the frequency of spontaneous excitatory postsynaptic currents (sEPSCs) through TRPV1 channels (Musumeci et al., 2011; Rossi et al., 2012) and that it blocked the capability of CB1 receptors in the control of glutamate transmission (De Chiara et al., 2013). Furthermore, IL-1β plays a crucial role in the induction of COX-2, 1 and PGE2 (Fiebich et al., 2000). Non-steroidal anti-inflammatory drugs (NSAIDs) inhibiting COX-activity, were able to decrease the inflammatory effects of IL-1β in the brain (Favrais et al., 2007). We show here a tendency of decreased COX-2 levels by AM404, whereas mPGES-1 expression was not affected. In our previous study, corroborating with our findings, we demonstrated that AM404 slightly decreased the levels of LPS-induced COX-2 protein but not of LPS-induced mPGES-1 (Saliba et al., 2017a).

The production of inflammatory mediators is mainly modulated by the neuroimmune cells, microglia, and astrocytes. IL-1β expression in microglia and astrocytes were increased in the cortex and striatum of rats after NMDA-induced excitotoxicity (Pearson et al., 1999). Subsequently, we verified the expression of cellular markers of microglia and astrocytes after NMDA stimulation and pre-treatment with AM404. NMDA stimulation statistically increased the expression of both markers, but AM404 did not alter these effects. In one of our previous studies using a mouse model of quinolinic acid (QA)- induced excitotoxicity pre-treated with rapamycin, we also observed no correlation between the alterations on cytokine expression and the activation of microglia or astrocytes (Saliba et al., 2017b).

Undoubtedly, further studies are still necessary to understand the role of AM404 during excitotoxic events and, additionally, to understand if the effects observed involve the activation of TRPV1 and CB1 receptors.

## Conclusions

In conclusion, we provide direct evidence that AM404 modulates the two major processes involved in neurodegenerative diseases, excitotoxicity and neuroinflammation, by decreasing pro-inflammatory mediators, reducing glutamate release, and calcium ions responses.

To understand the effects of AM404 in these processes might contribute to the therapeutic potential of AM404 in diseases with involvement of neuroinflammation and neurodegeneration and might lead to a possible future treatment of neurodegenerative diseases. However, further pre-clinical and clinical experiments in humans are necessary to evaluate other pharmacological parameters and safety of AM404 for further drug development.

## Data Availability Statement

All datasets generated for this study are included in the article/Supplementary Material.

## Ethics Statement

The animal study was reviewed and approved by Regierungspräsidium Freiburg (protocol No. X-13/06A), and carried out in accordance with the German animal welfare law for the use of experimental animals.

## Author Contributions

SS, TB, TS, AO, and BF participated in the research design. The experiments were performed by SS, TB, and TS. Data were analyzed by SS and TB. SS, TB, TS, GB, AO, and BF wrote or contributed to the writing of the manuscript. In addition, SS, TB, TS, GB, AO, and BF reviewed the data and discussed the manuscript. All authors have read and approved the final version of the manuscript.

## Conflict of Interest

The authors declare that the research was conducted in the absence of any commercial or financial relationships that could be construed as a potential conflict of interest.

## Supplementary Material

FIGURE S1 Effects of NMDA on levels of inflammatory mediators in OHSC. The OHSCs were stimulated with LPS or NMDA (10–50 μM). After 4 h, IL-1β **(A)**, IL-6 **(B)**, TNFα **(C)**, mPGES-1 **(D)**, and iNOS **(E)** were measured by qPCR. After 24 h, COX-2 **(F)** was evaluated by western blot. Data are expressed as mean ± SEM of at least three OHSCs/group. **p* < 0.05, ***p* < 0.01 and ****p* < 0.001 with respect to negative control (one-way ANOVA followed by the Newman–Keuls post-test).
