# Supplementary figures and images for: Neuroprotective Effect of AM404 Against NMDA-Induced Hippocampal Excitotoxicity
Source: Front Cell Neurosci. 2019 Dec 20;13:566. doi: 10.3389/fncel.2019.00566 (PMC6932953; doi:10.3389/fncel.2019.00566)

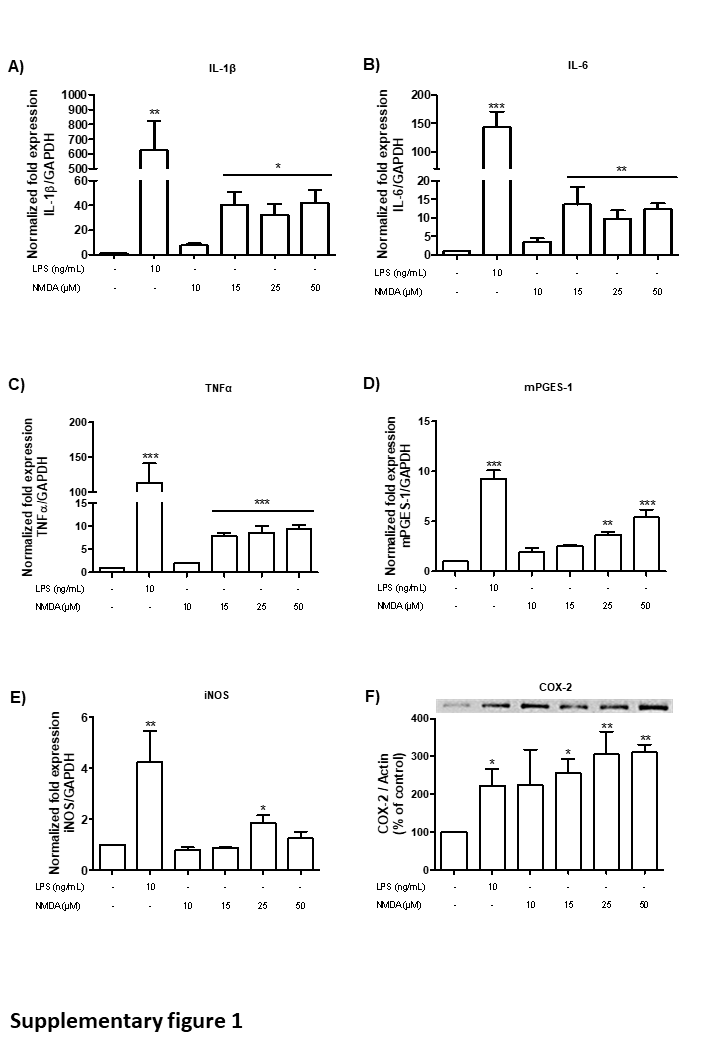

Supplement: FIGURE S1 — Effects of NMDA on levels of inflammatory mediators in OHSC. The OHSCs were stimulated with LPS or NMDA (10–50 μM). After 4 h, IL-1β (A), IL-6 (B), TNFα (C), mPGES-1 (D), and iNOS (E) were measured by qPCR. After 24 h, COX-2 (F) was evaluated by western blot. Data are expressed as mean ± SEM of at least three OHSCs/group. *p < 0.05, **p < 0.01 and ***p < 0.001 with respect to negative control (one-way ANOVA followed by the Newman–Keuls post-test). [file Image_1.TIF]
